# Quantification of active and total transforming growth factor-β levels in serum and solid organ tissues by bioassay

**DOI:** 10.1186/1756-0500-5-636

**Published:** 2012-11-14

**Authors:** Shaukat A Khan, Jennifer Joyce, Takeshi Tsuda

**Affiliations:** 1 Nemours Biomedical Research, Alfred I. duPont Hospital for Children, 1600 Rockland Rd, Wilmington, DE, 19803, USA; 2 Nemours Cardiac Center, Alfred I. duPont Hospital for Children, 1600 Rockland Rd, Wilmington, DE, 19803, USA

**Keywords:** Transforming growth factor-β (TGF-β), Βioassay, Plasminogen activator inhibitor-1, Mink lung epithelial cells, Enzyme-linked immunosorbent assay (ELISA), Myocardial infarction, Ventricular remodeling, Biomarker

## Abstract

**Background:**

Transforming growth factor-β (TGF-β) is a multi-factorial peptide growth factor that has a vital role in the regulation of cell growth, differentiation, inflammation, and tissue repair. Quantification of biologically active TGF-β levels in tissues is crucial to illustrate mechanisms involved in various physiological and pathological processes, but direct measurement of bioactive TGF-β level in the tissue has been hampered by lack of reliable methods. Here, we introduced mink lung epithelial cell bioassay to quantify both active and total TGF-β levels in serum and protein lysates from solid organs in the mouse model.

**Findings:**

Mink lung epithelial cells were stably transfected with plasminogen activator inhibitor-1 promoter/luciferase construct, in which bioactive TGF-β level was represented by luciferase activity. Serum total TGF-β levels were comparable between the bioassay and enzyme-linked immunosorbent assay (ELISA), but active TGF-β levels measured by ELISA were significantly lower than those obtained by the bioassay. Active and total TGF-β levels in the solid organs including heart, liver, and kidney were also measured. Total TGF-β levels were relatively comparable among these organs, but active TGF-β levels were slightly higher in hearts and kidneys than in livers. Positive luciferase activities in the bioassay were almost completely inhibited by adding pan-TGF-β neutralizing antibodies, suggesting its high specificity to bioactive TGF-β. We also measured myocardial TGF-β levels after myocardial infarction and sham control by the bioassay, and compared the values with those obtained by ELISA. The bioassay demonstrated that both active and total tissue TGF-β levels were significantly higher in post-myocardial infarction than in sham myocardium. ELISA was markedly less sensitive in detecting both active and total TGF-β levels than our bioassay and failed to show any statistically significant difference in TGF-β levels between myocardial infarction and sham myocardium.

**Conclusions:**

Our data suggested that the bioassay was significantly more sensitive than ELISA in detecting active TGF-β in serum and both active and total TGF-β in solid organ tissues. The bioassay will be useful in investigating TGF-β profile in various solid organs in physiological and pathological conditions.

## Findings

### Background

The transforming growth factor-β (TGF-β) superfamily consists of more than 40 members including TGF-β, activins, inhibins, growth differentiation factors, and bone morphogenetic proteins. They regulate a variety of physiological processes, including embryonic development, cell proliferation, differentiation, migration, adhesion, extracellular matrix (ECM) production, chemotaxis, homeostasis, and wound healing [1-7]. Three TGF-β isoforms, TGF-β1, TGF-β2, and TGF-β3, are known. The regulatory mechanisms of TGF-β activation process are complex (reviewed in [8-10]). The TGF-β is synthesized as an inactive protein, latent TGF-β, which consists of TGF-β dimer, and a latency-associated peptide (LAP). This small latent protein complex binds with the latent TGF-β binding proteins (LTBP) and is anchored in the insoluble ECM. The TGF-β is activated when a large protein complex is released from ECM via proteolytic cleavage. Dysregulation of the TGF-β pathway has been implicated in many human diseases including neoplastic [8,11], fibrotic [2,9], and cardiovascular diseases [10], but TGF-β involvement in these pathological conditions is complex and sometimes paradoxical [11,12].

Several methods have been developed and utilized to assess TGF-β profile in the tissue. Tissue TGF-β profiles can be assessed by mRNA and protein levels using real-time reverse transcriptase polymerase chain reaction (qRT-PCR) and immunoblots, respectively. Other approaches include nuclear localization of TGF-β or pSmad protein by immunohistochemical assay [13,14], enzyme-linked immunosorbent assay (ELISA) [15,16], and plasminogen activator inhibitor (PAI)-1 promoter/luciferase assay using mink lung epithelial cells (MLEC) [17,18]. Quantification of TGF-β levels in biological fluids including plasma, serum, and saliva has been reported; whereas, reliable measurement of TGF-β in solid organ tissues has been challenging because a substantial amount of TGF-β is sequestrated in insoluble ECM. Previously, ELISA has been used to measure total TGF-β1 levels in some human tissues [15,16]. However, ELISA is limited in its effectiveness of antigen-antibody response being subject to multiple environmental parameters and its inability to measure biologically active TGF-β.

In this study, we used a bioassay in which MLECs were stably transfected with an expression construct containing a truncated PAI-1 promoter fused to the firefly luciferase reporter gene [17,18] to quantify both biologically active and total TGF-β in serum, liver, heart, and kidney tissue. We also compared TGF-β levels measured by MLEC bioassay and ELISA in serum and myocardial tissues. Our results demonstrated that MLEC bioassay was a very specific and highly sensitive method to detect myocardial TGF-β levels when compared with commercially available ELISA. In addition, we found that serum TGF-β levels, even in normal control mice, were markedly higher than previously reported [19,20] and that only a small fragment of total serum TGF-β (approximately 1.5%) was biologically active. These findings suggest that TGF-β is not only a local growth factor but also plays a systemic endocrine role in regulating tissue homeostasis and that the serum also serves as a large reservoir for latent TGF-β in addition to tissue extracellular matrix.

### Materials and methods

#### Reagents

The purchased reagents include human recombinant TGF-β1 and protease inhibitor cocktail (Roche Diagnostic, Indianapolis, IN), Tris Base, sodium fluoride and sodium ortho vanadate (Fisher Scientific, Pittsburgh, PA), sodium pyrophosphate (decahydrate), sodium molybdate, and sodium glycerophosphate (Sigma-Aldrich, St. Louis, MO), and TGF-β1, 2, and 3 neutralizing antibody and TGF-β1 ELISA kit (both R&D Systems, Minneapolis, MN).

#### Cell culture and transfection

Mink lung epithelial cells (ATCC, Manassas, VA) were cultured in minimum essential medium (MEM) (ATCC, Manassas, VA) supplemented with 10% fetal bovine serum (Invitrogen Life Technologies, Carlsbad, CA). Mink lung epithelial cells are rapidly growing cells requiring splitting (1:5) every two to three days. These cells (7×10^5^ cells) were seeded in a 10-cm dish. After 24 hours, cells were transfected with PAI-1 promoter-Luciferase construct (generously provided by Dr. D.B. Rifkin, New York University Medical Center) [21] by FuGENE-6 transfection reagent (Roche Diagnostic, Indianapolis, IN) according to the manufactures’ protocol. Once the MLECs became confluent, cells were split and grown in the presence of 400 μg/ml of Geneticin (Invitrogen, Life Technologies, Carlsbad, CA) for stable clone selection. Only transfected cells can survive in the presence of Geneticin because the expression construct contains neomycin-resistant gene. Twenty-four neomycin-resistant colonies were picked and were separately grown in a 24-well plate with Geneticin. These clones were expanded to a 12-well plate, followed by a a 6-well plate, then T25 flasks, and finally T75 flasks in presence of 400 μg/ml Geneticin. The clones were trypsinized, resuspended in cryo medium (complete media plus 5% dimethyl sulfoxide [DMSO]), and kept in liquid nitrogen. After testing the clones for luciferase activity with human recombinant TGF-β1, clone #22 was found most sensitive and was thus used in all the ongoing experiments in presence of 250 μg/ml of Geneticin. The clone was used up to a maximum of 30 passages.

#### Animals and experimental myocardial infarction

For measurement of TGF-β in serum and various solid tissue organs including liver, heart, and kidney, C57BL/6 male mice 8 weeks of age were used. After mice were euthanized with excessive CO_2_ inhalation, the blood, liver, heart, and kidney were harvested under sterile conditions. The blood samples were kept on ice and the serum was isolated by centrifuge. For the MI study, C57BL/6 male mice of 12 to 20 weeks old were used. Non-reperfused experimental MI was performed by procedures described previously [22]. Briefly, under isoflurane anesthesia (2%), the heart was exposed via small left thoracotomy and suture ligation was placed immediately distal to the bifurcation of the left anterior descending artery with 6.0 silk sutures. Sham operation was performed with the suture insertion at the same site of the left ventricular free wall without suture ligation. After two weeks, non-ischemic left ventricular myocardium was dissected from post-MI and sham-operated mice upon euthanization with excessive CO_2_ inhalation. All animal procedures in this study were performed in adherence with the National Institutes of Health Guidelines on the Use of Laboratory Animals and were approved by the Institutional Animal Care and Use Committee (IACUC) of the Alfred I. duPont Hospital for Children.

#### Preparation of tissue extract

Isolated solid tissues were rinsed quickly in a sterile normal saline to remove the blood and were briefly placed on a sterile cloth to let dry. The collected organs were cut in small pieces, kept in cryo tubes on dry ice, and subsequently stored at −70°C until further usage. To obtain tissue lysates, approximately 30 to 50 mg of tissues were minced and sonicated in 500 μl of lysis buffer (50 mM Tris–HCl pH 7.5) containing 100 mM sodium fluoride, 30 mM sodium pyrophosphate, 2 mM sodium molybdate, 1 mM sodium ortho vanadate, 1 mM glycerophosphate, and 1x protease inhibitor cocktail on ice. Samples were centrifuged at 13,000 rpm for 20 minutes at 4°C. Clear supernatant was collected and stored in aliquots at −70°C. Protein quantification in the lysate was done by the bicinchoninic acid (BCA) method [23].

#### Acid activation of serum and tissue extracts

To assess the amount of total TGF-β, acid activation was performed with minor modification to isolate free TGF-β molecules from latent complex [18]. Briefly, 30 μl of serum or protein lysate (equivalent to 100 to 300 μg protein) was added to 200 μl of MEM/bovine serum albumin (BSA), followed by addition of 10 μl of 4 N HCl. Samples were rocked for 1 hour at 4°C. Acid activation was stopped by neutralization with 10 μl of 4 N NaOH. Once acid activated, the samples were stored on ice and were used on the same day.

#### Bioassay with MLECs

After being treated with trypsin, MLECs (4 ×10^4^ cells) were plated into a 96-well plate and incubated for three hours at 37°C. First, different concentration (25 pg/ml to 100 pg/ml) of human recombinant TGF-β1 solutions in 200 μl of DMEM/BSA containing 250 μg/ml Geneticin was plated in triplicates to obtain a standard luciferase activity in response to bioactive TGF-β1. For active TGF-β assay, 30 μl of serum samples were diluted with 220 μl of MEM/BSA (total 250 μl), which was further diluted two times with MEM/BSA to a final volume of 500 μl. Twenty μl of these diluted serum samples were again diluted with MEM/BSA containing 250 μg/ml Geneticin to make a final volume of 200 μl, which were transferred to a 96-well plate in triplicate replacing the previous culture medium. The plate was incubated for 20 hours at 37°C and then harvested for luciferase assay. For total serum TGF-β assay, samples were first acid-treated as described above. Acid treated samples (250 μl) were further diluted to a final volume of 5 ml MEM/BSA to get luciferase reading in linear range. Twenty μl of these diluted serum samples were added to a 96-well plate in triplicate and final volume of the well was made 200 μl with MEM/BSA containing 250 μg/ml Geneticin. Tissue TGF-β bioassay was performed similar to serum. Instead of serum, 30 μl of tissue homogenate (equivalent to 100 to 300 μg protein) were added to 220 μl of MEM/BSA, which was further diluted up to a final 500 μl MEM/BSA. Twenty μl of the diluted sample was added to a 96-well plate as above. All samples were incubated for 20 hours. The tissue homogenate or serum samples were diluted differently so that the active and total TGF-β relative luciferase activity lies in the linear range of standard curve.

#### Luciferase assay

After trypsinization, MLECs were replated into a 96-well plate in 200 μl of MEM medium with 10% fetal bovine serum (FBS) and incubated for 3 h at 37°C. All samples, with or without acid activation, were incubated in MEM/BSA in triplicate for 20 h at 37°C. Then, the cells were harvested by adding 30 μl 1x passive lysis buffer (Promega, Madison, WI) after washed with 1x phosphate buffer saline (PBS). The plate was kept on rocker for five minutes and then stored at −70°C before luciferase assay. Luciferase assay reagent (100 μl: Promega, Madison, WI) was added to each well by injector after being thawed in a 37°C water bath and kept on rocker for five minutes. The relative luciferase unit (RLU) was read by 2030 Multilabel Reader (Perkin Elmer, Waltham MA). Corresponding TGF-β levels were calculated by subtracting the RLU of control from the RLU of study samples because MLEC itself has endogenous TGF-β activity. The TGF-β concentration was calculated with a standard curve using straight line formula. The serum TGF-β was represented as ng/ml, whereas the tissue TGF-β was presented as ng/mg.

#### Inhibition of TGF-β by pan TGF-β neutralizing antibody

To test the specificity of PAI-1 promoter/luciferase response to bioactive TGF-β, we measured luciferase levels of the serum and liver homogenate with and without TGF-β neutralizing antibody. Either 30 μl of serum or liver homogenate (100 μg) was incubated with or without 100 μg/ml TGF-β 1, 2 and 3 neutralizing antibody in a final volume of 200 μl of MEM/BSA for two hours at 4°C while rocking followed by acid activation as described in the previous section. These samples were then used in a 96-well plate containing MLECs for luciferase activity as described above.

#### Statistics

Graph-Pad Prism 5 software (GraphPad Software, San Diego, CA) was used to compare the groups. One-way analysis of variance with Newman-Keuls post test or two tailed paired t-test were used to assess the significance of data values. A *p* value of less than 0.05 is considered as significant. All of the values were shown as mean ± standard deviation unless indicated otherwise.

### Results

#### Transforming growth factor β (TGF-β) assay using human recombinant TGF-β1

**Figure 1:**
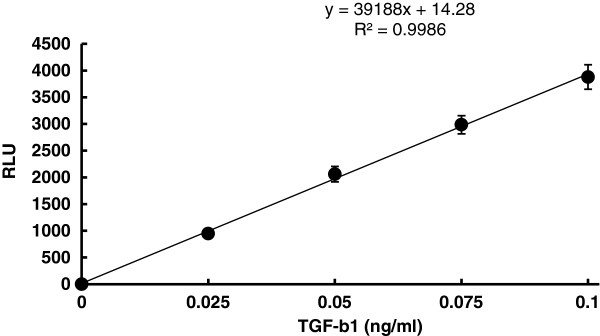
**Human recombinant TGF-β1 (hTGF-β1) - luciferase standard curve.** PAI-1 promoter/luciferase construct-transfected MLECs were incubated with various concentration of human recombinant TGF-β1 at 37°C for 20 h. There is a strict dose-dependent increase in luciferase activity (RLU) by TGF-β1 between 0 and 0.1 ng/ml. The values represents mean ± SD of 5 individual experiments. MLECs=mink lung epithelial cells, PAI=plasminogen activator inhibitor, RLU=relative luciferase unit, SD=standard deviation, TGF-β=transforming growth factor-β.

In this study, we created new MLEC cell lines by transfecting PAI-1/luciferase construct *de novo*. First, the luciferase assay was performed with different concentrations of highly pure (> 95%) human recombinant TGF-β1 to obtain a standard dose-responsive curve. A consistent linear dose-dependent relationship was obtained repeatedly at TGF-β1 concentration from 0 to 100 pg/ml corresponding 0 to 4000 RLU, at which range we used to quantify TGF-β concentration (Figure 1). This linear relationship was not consistent over 100 pg/ml. A standard curve was obtained at each experiment to simultaneously quantify TGF-β concentration.

#### TGF-β levels in serum

**Figure 2:**
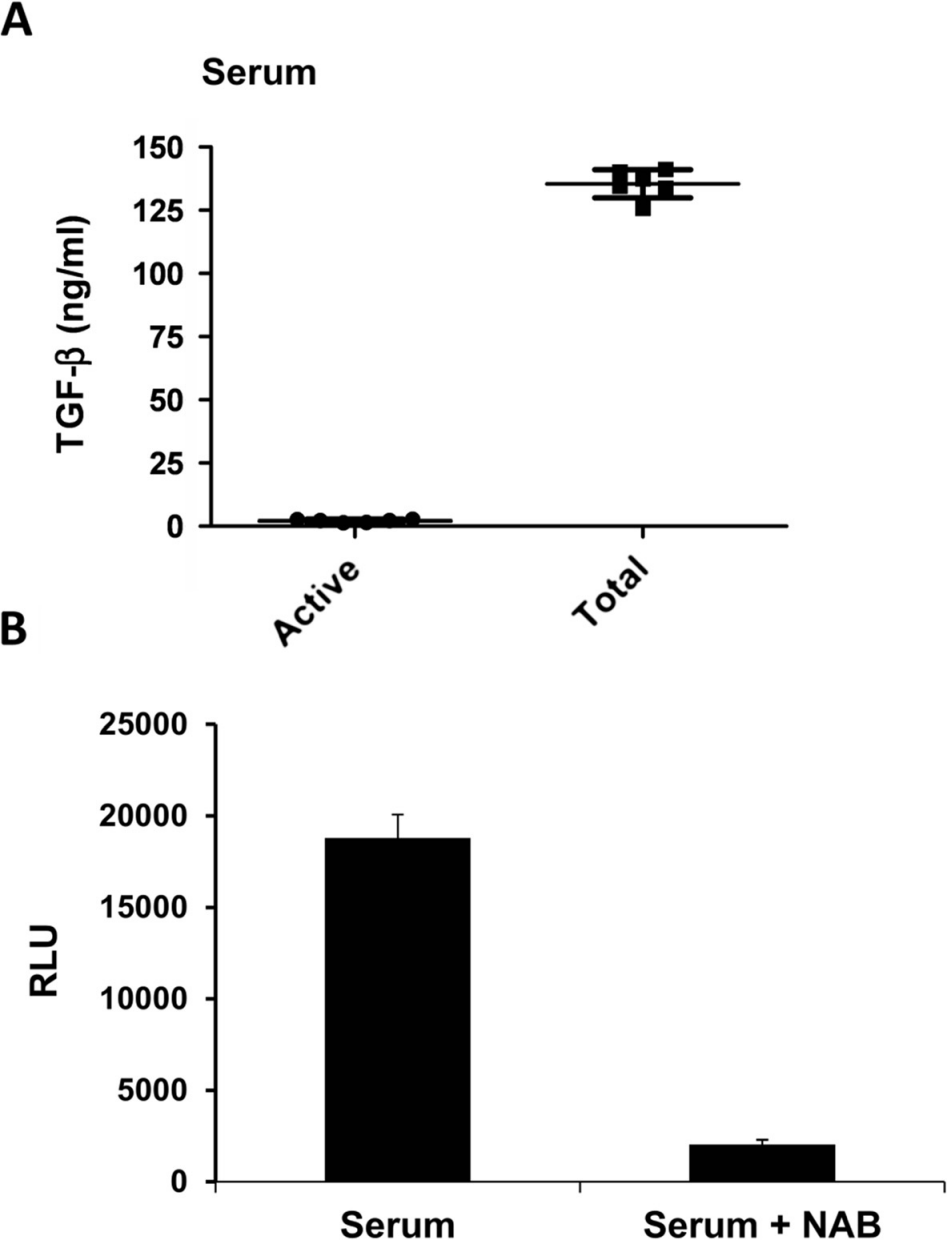
**Active and total TGF-β levels in serum and inhibition of serum TGF-β by pan TGF-β neutralizing antibodies. A**. Active and total TGF-β levels in serum. **B** Inhibition of total serum TGF-β in the absence or presence of 100 μg/ml of pan TGF-β neutralizing antibodies. RLU was inhibited by 92% with neutralizing antibody. Sample size: n = 6. NAB=neutralizing antibodies, RLU=relative luciferase unit, TGF-β=transforming growth factor-β.

It is known that TGF-β binds to a carrier protein, α2-macroglobulin, in a non-covalent manner as a latent form in the blood stream [24,25]. Active (= free) and total (= free + latent) serum TGF-β levels were 2.1 ± 0.58 ng/ml and 136 ± 5.59 ng/ml, respectively, suggesting that the level of the latent form of TGF-β is approximately 65 times higher than the active form in the blood stream. In addition, we found that the total serum TGF-β level was markedly higher than what was reported previously [19,20] (Figure 2A). The specificity of this bioassay was previously confirmed in human and rat plasma [18]. We also tested the specificity of the bioassay in this newly-created cell line. The inhibition of TGF-β-induced RLU response in the serum was examined by adding TGF-β1, 2, and 3 neutralizing antibodies to the sample, as previously described. By adding pan TGF-β neutralizing antibodies, the measured serum TGF-β levels were inhibited by 92%, suggesting its high specificity to bioactive TGF-β (Figure 2B).

#### TGF-β bioassay in liver homogenate

**Figure 3:**
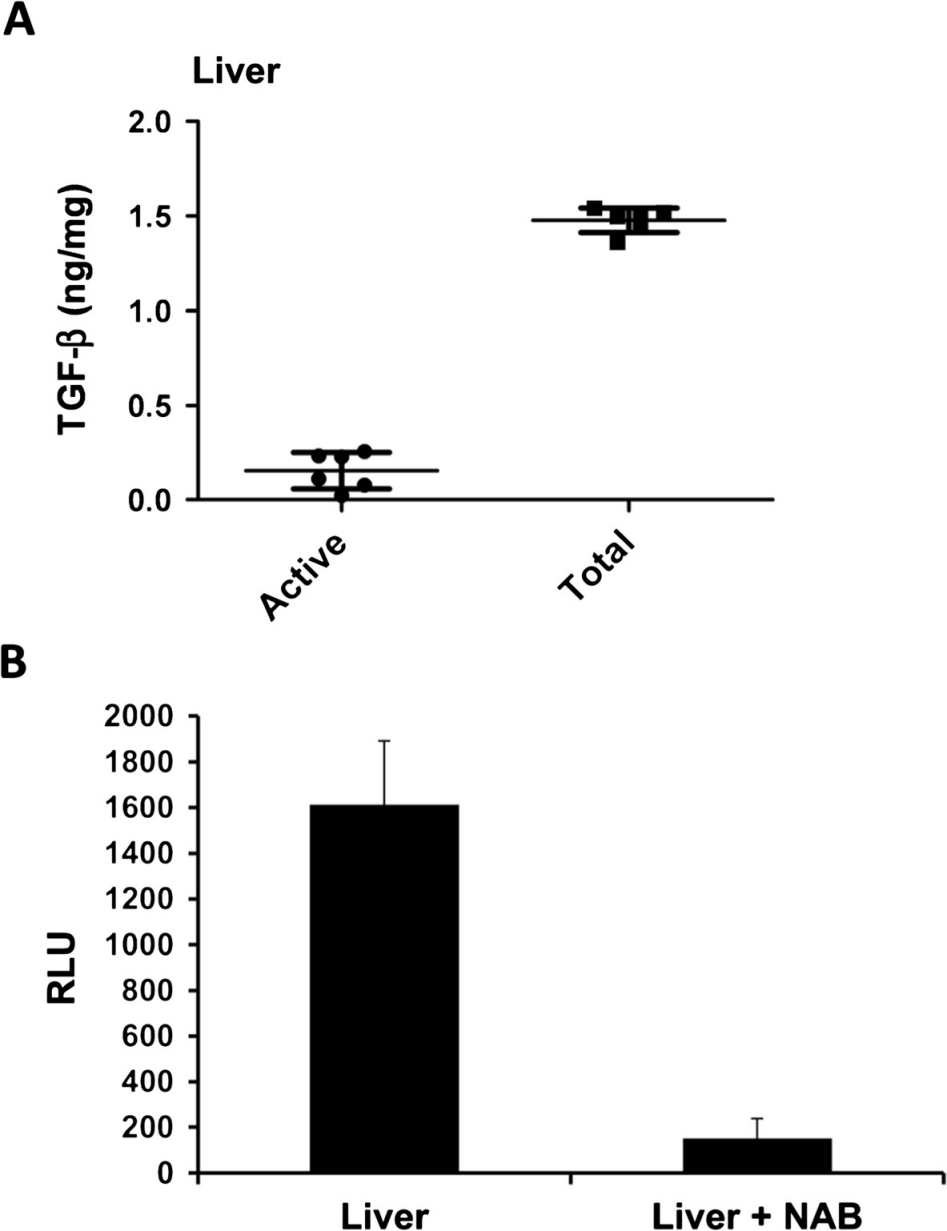
**Active and total TGF-β levels in liver homogenate and inhibition of liver TGF-β by pan TGF-β neutralizing antibodies. A**. Active and total TGF-β levels in liver. **B** Inhibition of total liver TGF-β in the absence or presence of 100 μg/ml pan TGF-β neutralizing antibody. TGF-β level was 97% inhibited by neutralizing antibody. Sample size: n = 6. NAB=neutralizing antibodies, RLU=relative luciferase unit, TGF-β=transforming growth factor-β.

We next measured active and total TGF-β levels in the liver homogenate. The active and total TGF-β levels in liver homogenate were 0.15 ± 0.10 ng/mg and 1.48 ± 0.07 ng/mg, respectively. In the liver, the total TGF-β level was about 10-fold higher than the active TGF-β level (Figure 3A). The specificity of the bioassay to hepatic TGF-β was also tested by measuring how much luciferase activity was inhibited by adding TGF-β1, 2 and 3 neutralizing antibodies to the protein lysate of the liver, as explained in the previous section. Again, luciferase activity was inhibited by 97% by the neutralizing antibodies, suggesting its high specificity even for hepatic TGF-β (Figure 3B).

#### TGF-β bioassay of heart and kidney homogenates

**Figure 4:**
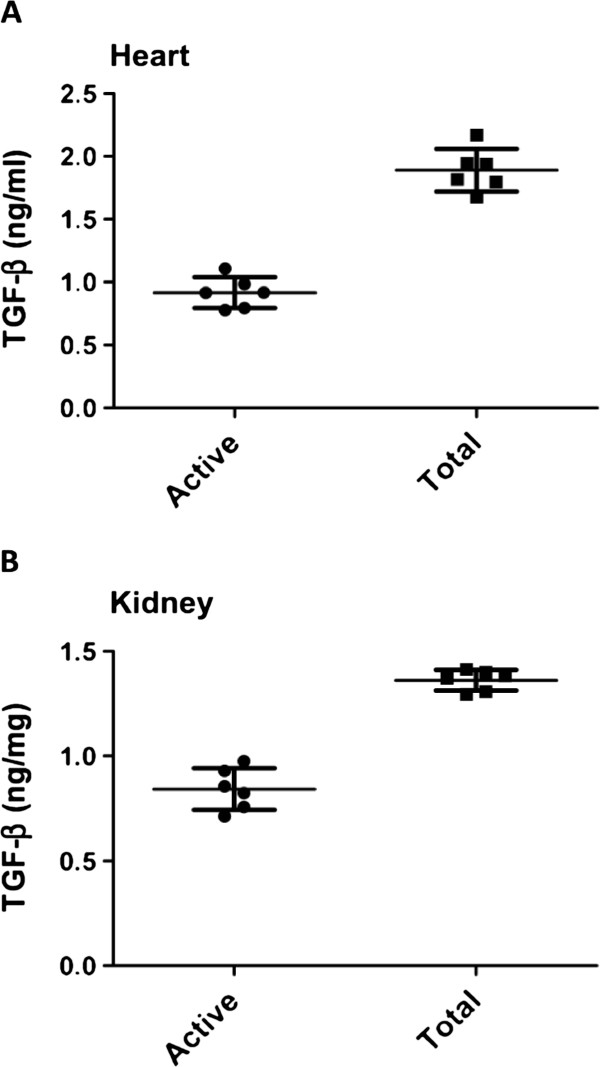
**Active and total TGF-β levels in heart and kidney.** Active and total TGF-β levels in heart homogenate (**A**) and kidney homogenate (**B**). Sample size: n = 6. TGF-β=transforming growth factor-β.

Both active and total TGF-β levels in heart homogenate were 0.92 ± 0.12 and 1.89 ± 0.17 ng/ml, respectively. The total myocardial TGF-β was approximately two-fold higher than the active form, as shown in Figure 4A. Active and total TGF-β levels in kidney homogenate were 0.84 ± 0.10 and 1.36 ± 0.05 ng/mg, respectively. The total TGF-β level in the kidney was only 60% higher than the active form (Figure 4B). In the three different solid organ tissues (liver, heart, and kidney), total TGF-β levels were relatively comparable; whereas, the active form differed slightly among the organs.

#### Comparison of TGF-β levels measured by ELISA versus MLEC bioassay in serum and heart

To compare the efficacy of our MLEC bioassay with that of a commercially available ELISA kit (R&D Systems, Minneapolis, MN), we measured both active and total TGF-β levels in serum and in heart by the two methods. The heart tissues include post-myocardial infarction (MI) non-ischemic ventricular myocardium and that of sham-operated mice at two weeks after an experimental coronary artery ligation. The TGF-β is known to be up-regulated after MI and is thought to play a principal role in post-MI ventricular remodeling [26]. We examined whether tissue TGF-β levels were increased in post-MI left ventricular myocardium remodeling compared with the sham operated hearts.

**Figure 5:**
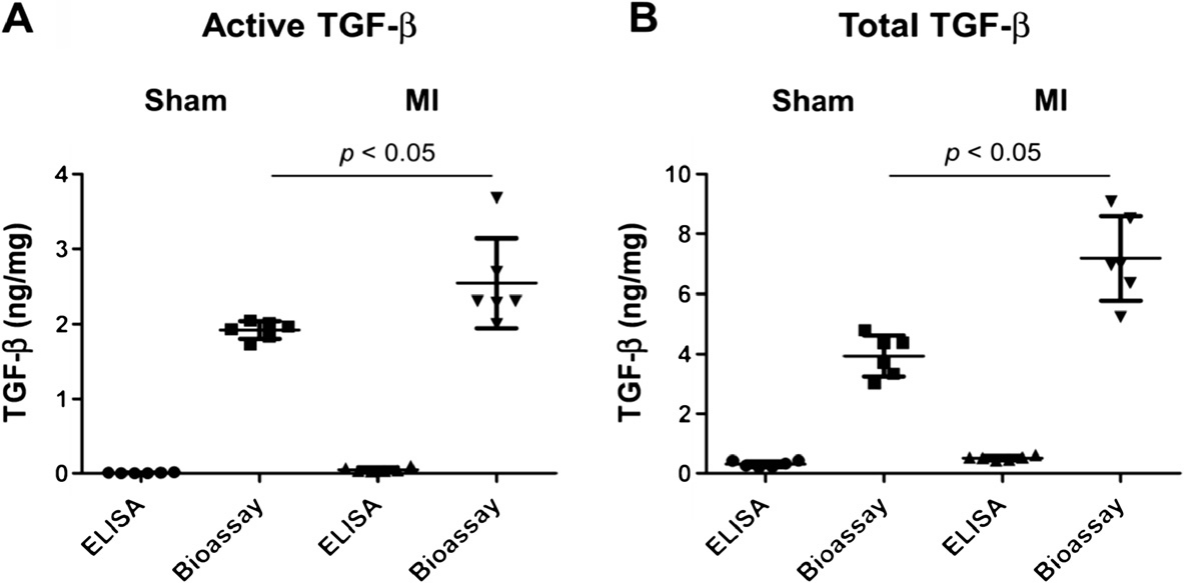
**Active and total TGF-β levels in sham and two weeks post-myocardial infarction (MI) mice left ventricular myocardium measured by ELISA and bioassay.A**: Active TGF-β levels and **B**: Total TGF-β levels in sham and two weeks post-MI heart homogenate. For both sham and MI, bioassay revealed not only significantly higher TGF-β levels than ELISA (*p* < 0.05), but also showed significant increase of both TGF-β levels in MI compared with sham (*p* < 0.05), whereas ELISA failed to show significant increase in MI compared with sham. Sample size: n = 6. ELISA=enzyme-linked immunosorbent assay, MI=myocardial infarction, TGF-β=transforming growth factor-β.

First, ELISA revealed comparable total TGF-β levels with bioassay in serum (ELISA 138 ± 12 ng/ml, bioassay 135 ± 5.6 ng/ml) but showed significantly lower active TGF-β levels in serum than bioassay (ELISA 0.92 ± 0.45 ng/ml, bioassay 2.1 ± 0.6 ng/ml). Secondly, both active and total TGF-β levels in myocardial tissues by ELISA were markedly lower than those by bioassay. By bioassay, the active myocardial TGF-β levels in sham and MI mice were 1.92 ± 0.12 ng/mg and 2.54 ± 0.59 ng/mg (*p* = 0.039), respectively. The total myocardial TGF-β levels in sham and MI mice were 3.94 ± 0.69 ng/mg and 7.19 ± 1.41 ng/mg (*p* = 0.0087), respectively (Figure 5). ELISA showed extremely low yield in both active (sham 0.00540 ± 0.00672 ng/mg; MI 0.0498 ± 0.0302 ng/mg) and total TGF-β levels (sham 0.312 ± 0.096 ng/mg; MI 0.510 ± 0.066 ng/mg) and failed to demonstrate any significant increase of either active or total TGF-β levels in the post-MI myocardium compared with the sham myocardium. Our data illustrated that ELISA showed comparable sensitivity with MLEC bioassay only when measuring total serum TGF-β levels, but that ELISA showed exceedingly low yield in detecting both active and total TGF-β in the myocardial tissue than did MLEC bioassay.

### Discussion

The purpose of this study was to measure TGF-β levels in the serum and solid organ tissues of mice, including myocardium after MI in comparison with sham. To do this, we developed sensitive reporter assay using MLECs. By using this bioassay, we were able to measure both active and total TGF-β levels in serum, liver, heart, and kidney tissues. Especially for active TGF-β, MLEC bioassay revealed much better sensitivity than ELISA not only in solid organ tissues but also in serum. It is extremely advantageous to know both active and total TGF-β levels simultaneously to better delineate how TGF-β is involved in ordinary physiological conditions and pathogenesis of various progressive disease processes.

This bioassay is not a new method but rather a well-established method that has been used to measure TGF-β in biological fluids and rat lung [17,18], but it has not been applied to measure TGF-β levels in solid organs. It is surprising that this method has not been widely used over decades; recently, many investigators have used ELISA to measure total TGF-β levels in serum and solid organ tissues. We have slightly modified the original method and measured TGF-β levels in serum as well as solid organ tissues. Our current modification includes a newly transfected MLEC cell line, a narrower range of standard dose-responsive relationship, and use of a more purified recombinant TGF-β to create a more stringent standard dose-responsive curve. Direct measurement of TGF-β in solid organs has been challenging because of complex involvement of TGF-β in both soluble and insoluble forms. The known available methods to assess active TGF-β include semi-quantitative methods such as using transgenic mice-containing green fluorescent protein under the control of TGF-β responsive element (CAGA)_12_[21,27], using specific antibodies to bioactive TGF-β [28-30], or indirect measurements by antibodies that recognize pSmad2 and pSmad3 [31-33]. Nevertheless, the direct measurement of bioactive TGF-β in the tissues still remains a major challenge.

We took advantage of the MLEC bioassay system and selected the standard curve in a very narrow range from 0 to 100 pg/ml in which biologically active TGF-β binds to PAI-promoter to activate luciferase in a strict dose-dependent manner. Commercially available ELISA are commonly used to measure total TGF-β levels mostly in serum, plasma, urine, and other biological fluids [34,35], and sometimes in solid tissues [15,16,34]. Our data showed that the sensitivity of ELISA and bioassay was equal when measuring total serum TGF-β levels, but the bioassay showed markedly higher sensitivity in both active and total TGF-β levels in tissues than ELISA. At the same time, this bioassay was also proven to be highly specific (Figures 2B and 3B). The advantage of ELISA is its wide measurement range, whereas the disadvantage is that the biological activity of the detected antigen is uncertain, especially for active TGF-β [21] and poor sensitivity when measuring solid organ tissues. The major advantage of this bioassay is its ability to measure both active and total TGF-β in serum and solid organs. Understanding both active and total TGF-β levels simultaneously would provide us with further insight into how TGF-β is involved in physiological and pathological conditions including chronic fibrotic disorders [2,9], heart failure [36-38], and cancer progression [8,11].

Our current study also indicated that circulating blood or serum serves as an enormous reservoir for bioactive TGF-β. In this study, we elected to use serum instead of plasma because plasma contains platelets, another major source of TGF-β. The TGF-β levels in serum and platelet-poor plasma in humans are known to be comparable [19]. Our results not only demonstrated that the circulating total TGF-β level was markedly higher than what was reported previously [19,20] but also showed that the majority of serum TGF-β exists as a latent form with only a fraction being biologically active (1.6%). The presence of latent complex as a predominant form of circulating TGF-β suggests the endocrine role of TGF-β [39]. The latent TGF-β complex consists of bioactive free TGF-β and its non-covalently binding the major serum protein, α2-macroglobulin [25]. Circulating TGF-β levels were noted to be elevated in multiple pathological conditions including breast cancer [40], non-small cell lung cancer [41], hepatocellular carcinoma [42], and dilated cardiomyopathy [43]. These TGF-β levels were all total values measured by ELISA, but there has been no study to address circulating bioactive TGF-β levels in either physiological or pathological conditions. With simultaneous measurement of active and total TGF-β levels by the MLEC bioassay, we will be able to obtain deeper insights into TGF-β biology and its involvement as a biomarker in pathogenesis of various disease processes.

### Conclusion

The MLEC bioassay is a useful and reliable method to quantify both active and total TGF-β levels in both serum and various solid organ tissues, and it can be used to address more accurate TGF-β profiles in various pathological conditions. Direct measurement of active TGF-β levels in the tissue has never been well established. Simultaneous measurement of both active and total TGF-β levels in various pathological specimens would help us understand the mechanism of progressive disorders including fibrotic diseases, chronic inflammatory disorders, heart failure, and cancers in clinical settings. Our current data also suggest that TGF-β plays a role not only as a local tissue growth factor but also as a circulating hormone in both physiological and pathological conditions. Our current method will open an exciting new arena in studying TGF-β as an important biomarker in numerous clinical situations.

## Abbreviations

BCA: Bicinchoninic acid; BSA: Bovine serum albumin; DMSO: Dimethyl sufloxide; ECM: Extracellular matrix; ELISA: Enzyme-linked immunosorbent assay; FBS: Fetal bovine serum; IACUC: Institutional animal care and use committee; LAP: Latency-associated peptide; LTBP: Latent TGF-β binding proteins; MEM: Minimum essential medium; MI: Myocardial infarction; MLEC: Mink lung epithelial cell; PAI: Plasminogen activator inhibitor; PBS: Phosphate buffer saline; qRT-PCR: Real-time reverse transcriptase polymerase chain reaction; RLU: Relative luciferase unit; SD: Standard deviation; TGF-β: Transforming growth factor-β.

## Competing interests

The authors declare that they have no competing interests.

## Authors’ contributions

SK carried out all the experiments, designed or contributed to the design of the experiments, and contributed to the preparation of the manuscript. JJ participated in most of the experiments and primarily took care of the animals used in this study. TT designed and supervised the majority of the experiments, critically analyzed the data, and contributed to the preparation of the manuscript. All authors read and approved the final manuscript.
